# Influence of Soft or Hard Floors before and after First Calving on Dairy Heifer Locomotion, Claw and Leg Health

**DOI:** 10.3390/ani5030378

**Published:** 2015-08-06

**Authors:** Christer Bergsten, Evgenij Telezhenko, Michael Ventorp

**Affiliations:** 1 Biosystems and Technology, Swedish University of Agricultural Sciences, S-230 53 Alnarp, Sweden; E-Mail: michael.ventorp@hushallningssallskapet.se; 2 Viking Genetics, S-532 94 Skara, Sweden; E-Mail: evtel@vikinggenetics.com

**Keywords:** dairy cow, flooring system, rubber floor, locomotion, claw disorder, leg lesion, claw conformation, weight and pressure distribution, prevention

## Abstract

**Simple Summary:**

In this study the effect of different flooring systems on locomotion, claw conformation, loading, claw- and leg disorders was assessed in heifers from one year before to one year after calving. After calving, heifers kept on alleys covered with rubber flooring were found to develop less lameness, fewer claw disorders of the sole horn and fewer leg lesions than those kept on concrete alleys. Recruitment heifers reared on soft deep straw bedding had fewer sole horn lesions and more overgrown claws before calving, but were more prone to severe sole horn lesions after calving, than those reared in cubicles with hard concrete floors.

**Abstract:**

Claw health, an important dairy cow welfare parameter, may be affected by early-life foot/leg stresses. To investigate this, groups of pregnant heifers were allocated to deep straw bedding (Soft) or cubicles (Hard), both with scraped concrete feeding alleys. After the grazing season, they were re-housed in cubicle systems, half on slatted concrete (Hard) and half on slatted rubber (Soft) alleys. Claw measurements, contact area and pressure distribution claw/flooring, claw disorders and leg lesions were recorded at the start and end of each housing season. Locomotion and leg lesions were also scored monthly after calving. Prevalence of sole haemorrhages was higher among pregnant heifers in cubicles than in deep straw. After calving, first-calvers on Hard floors had higher odds for lameness (OR = 3.6; *p* < 0.01), sole haemorrhages/ulcers (OR = 2.2; *p* < 0.05), white-line haemorrhages (OR = 2.8; *p* < 0.01) and leg lesions (OR = 2.6; *p* < 0.02) than those on Soft floors. Lowest prevalence and severity of sole and white-line haemorrhages (non-significant) in first-calvers was found in those on Soft floors and reared on Hard floors and the highest prevalence and severity on those on Hard floors reared on Soft floors. Soft flooring after calving is of most importance for healthy feet and legs.

## 1. Introduction

Pasture, to which the claws were adapted during evolution, is normally the best ground for claws and legs. It gives a balance between claw horn wear and growth and the claws are loaded in a natural way [1]. Moreover, grazing dairy cows possibly have free access to a comfortable lying surface, which facilitates lying and rising and allows the vast majority of leg lesions, such as hairlessness, swellings and ulcers, caused by suboptimal stalls to recover. Where pasture area is sufficiently large, cow manure is widely dispersed, animal hygiene is generally very good and claws escape attacks by bacteria, which are often connected with manure contamination of the feet and legs. In contrast, housed cows are forced to walk on hard concrete and manure-contaminated floors to compete for feed, milking and finding available resting area, and the stocking density is often high. Suboptimal dairy cow flooring is a major risk factor for impaired foot and leg health.

The expression “claw quality” is often used to describe the morphological, anatomical and physiological characteristics of the claw capsule [2]. Claw conformation has a close connection with the biomechanics of the claw and the locomotor apparatus. How weight is distributed within and between the claws and on pressure points is of particular importance as it can change the conformation of the claw, cause claw disorders and influence locomotion. Claw conformation has genetic components, which have been the subject of breeding for healthier feet [3,4]. Toe angle and rear leg posture have thus long been used as traits for progeny testing as possible indirect markers and as a measure to improve claw health. For example, cows with long toes and shallow foot angles are more likely to have claw problems [5]. Moreover, Manske *et al.* [6] revealed a correlation between abnormal claw shape and sole ulcers in an epidemiological study. However, it is still a topic for discussion whether altered claw conformation is a risk factor itself or an effect of a claw disorder.

It is well known that management changes around calving, *i.e.*, the transition period, are highly important for production diseases. The metabolic load of lactating cows is significantly greater than that of heifers, although actual gestation also increases the metabolic load. However, the transition period also includes stress from change of diet, social stress between animals, change of groups and change of housing [7,8]. This can initiate claw and leg disorders and lameness, affecting the lactating cow and impairing performance. Apart from metabolic disturbances, locomotor lesions are often related to biomechanical changes and trauma, indirectly caused by changed stance periods, with too short a lying period and consequently too long a standing period on uncomfortable flooring [9,10]. However, more recent research [11] suggests that lameness itself alters the lying time, especially the number of lying bouts in the transition time. Replacement heifers are often neglected, with suboptimal housing and management, until calving. They are often kept as simply and cheaply as possible on straw-bedded yards or outdoors before being moved to the tougher environment in the milking herd. It is not unreasonable to assume that feet and leg strains before first calving have a significant influence on the feet and leg health of first-calving heifers. Vermunt and Greenough examined heifers from 1–2 years of age in two different management systems (outdoors on soft ground or indoors on slatted concrete floors) and determined claw conformation [12] and hock angle [13] using several metrics and claw disorders, e.g., sole haemorrhages at four-week intervals [14]. The results showed differences in all traits between the two different management systems, which those authors attributed to genetic or environmental influences on conformation. They found a surprisingly high prevalence of sole haemorrhages in heifers, even those outdoors. Although no follow-up was done, they concluded that these lesions are likely to have long-lasting consequences. The main aim of present study was to evaluate to what degree the management system *per se* and changes between flooring system of the pregnant heifers affect the conformation (horn growth and wear) and disorders of the claws, leg lesions and the risk of lameness during their first lactation. In the herd studied, we were able to have two parallel groups with alternating flooring systems in an experimental design of pregnant, recruitment heifers during the housing season before calving and two parallel groups also during the housing season in their first lactation.

## 2. Hypothesis

Claw conformation, leg lesions and claw disorders in recruitment heifers affect claw disorders, leg lesions and locomotion during their first lactation. Soft flooring systems reduce trauma-related claw disorders and improve locomotion.

## 3. Materials and Methods

### 3.1. Animals and Housing

**Figure 1:**
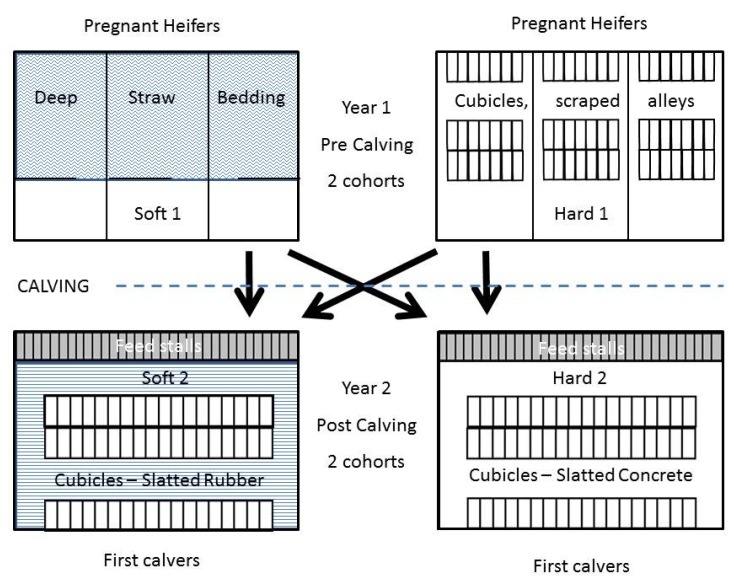
Schematic figure of the housing of pregnant heifers during Year 1 and the transition from Soft 1 pre calving to either Soft 2 or Hard 2 post calving during Year 2 and from Hard 1 pre calving to either Soft 2 or Hard 2 post calving during Year 2. The procedure was repeated for each of 2 cohorts. ~ = Deep straw bedding, = = Slatted rubber flooring, □ = Concrete flooring.

This study was performed on a commercial, organic 300-cow dairy in western Sweden comprising Swedish Red (SR, 52%), Swedish Holstein (SH, 39%) and crossbreeds (SR × SH, 9%, not used in the study), plus own recruitment heifers. The uninsulated 2 × 3 row cubicle house was constructed in 1995 and had a row of feed stalls (160 cm × 80 cm) separated by dividers equipped with rubber mats (UBO™), along the 40 cm elevated feeding platform. The alleys were made of slatted concrete (12.5 cm slat, 4 cm slot; Hard 2, Figure 1). In one compartment (Soft 2, Figure 1) the original concrete slats were covered with slatted rubber mats (KURA-S^®^, Kraiburg; 13 cm slat, 3.5 cm slot). Because of narrower openings and the reinforced mat construction, the drainage area was thereby reduced from 20% to 15%. All 285 cubicles were equipped with mattresses (KEW plus™, Kraiburg) and the partitions were attached at the front, without any support at the rear (Jyden, type 16K). Average milk yield during the three study years was 9300 kg energy-corrected milk (ECM) and all animals grazed outdoors from May to September as required by the Swedish organic association (KRAV). Young stock up to 12 months was kept in other buildings and from 12 months heifers were moved to a section in the same building as the milking herd.

The section used for recruitment heifers before calving in this study had two different layouts. On one side of the common feeding platform (Hard 1, Figure 1), there were three rows of cubicles (100 cm × 224 cm, in total 63). The concrete-based flooring was bedded twice weekly with a superficial layer of shavings and, during the second year, the cubicles were equipped with rubber mats (UBO™) plus a superficial layer of shavings. The concrete alleys (width 220 cm) between the cubicle rows and the feeding alley (width 360 cm) were automatically scraped twice daily with a mechanical scraper. On the other side of the feeding platform (Soft 1, Figure 1) there were three permanent, deep straw-bedded boxes (10 m × 10 m), which were bedded twice weekly during the whole housing season. A concrete alley (340 cm) in front of the boxes and along the feeding platform was automatically scraped twice daily. Space allowance (animal/m^3^) was the same in Hard 1 and Soft 1. The number of animals never exceeded the number of cubicles in any group (Swedish welfare legislation).

### 3.2. Experimental Design

The randomised 2 × 2 factorial study included two groups (cohorts) of dairy heifers, born in the second half of 2004 and 2005, respectively. The housing management during experimental Year 1 and Year 2 was repeated for each cohort of heifers. Between the housing periods all the heifers were grazed together. All heifers were reared similarly until the experiment started in their second housing season 2005 and 2006 (Year 1), when they had reached about one year of age (9–16 months). For each year, the heifers were randomly blocked into two equally sized groups according to breed and age, allocated to either cubicles (Hard 1) or deep straw-bedded boxes (Soft 1) during the winter housing period. The second part of the experiment started when the heifers were re-housed before their first calving, during the second half of 2006 and 2007 (Year 2), respectively. The pregnant heifers were randomly blocked according to previous treatment (Hard 1 or Soft 1), breed (SR, SH) and expected calving, and allocated to either cubicles with slatted concrete floors (Hard 2) or cubicles with slatted rubber mats (Soft 2). Heifers with expected calving before 15 November were then directly housed in the cubicle system described above, while those with expected calving after 15 November were housed in their previous heifer system until three weeks before expected calving. At that point, they were then grouped together with low-yielding cows in the same cubicles and slatted concrete flooring as in year 1 (2006) or slatted rubber mats as in year 2 (2007). Calving was supervised in separate straw-bedded boxes (3 m × 3 m) and the calf stayed with the cow for four days (organic legislation), after which the first-calving heifer entered her experimental group (Hard 2 or Soft 2). All management and feeding was the same for all animals, except for routines related to the specific system. This layout lasted through two housing seasons for each cohort of animals and terminated after their first lactation in 2007 and 2008, respectively.

### 3.3. Claw and Leg Observations

Claw conformation (growth and wear), claw disorders and leg lesions were assessed in association with trimming at the start of the experiment when the heifers were housed (Year 1), after their first housing season before grazing (Year 1), after housing and at first calving (Year 2) and at the end of experiment after their first lactation, before grazing (Year 2).

At the first examination at the study start (Year 1), a mark was branded in the toe wall of the outer rear claw, 35 mm from the tip of the toe. This mark was used as reference for growth and wear after 4 months of exposure to any flooring system, according to Graunke *et al.* [15]. After autumn and before spring trimming, the left rear claw of heifers and first-calving heifers was digitally photographed (Pentax Optio WP). Measurements were made from the digital pictures with the program UTHSCSA ImageTool (University of Texas, Health Science Center, San Antonio), which can be downloaded free from the internet. A verniper caliper was used to identify the anatomical reference for the measurements (perioplium) and for individual spatial calibration of each picture.

**Figure 2:**
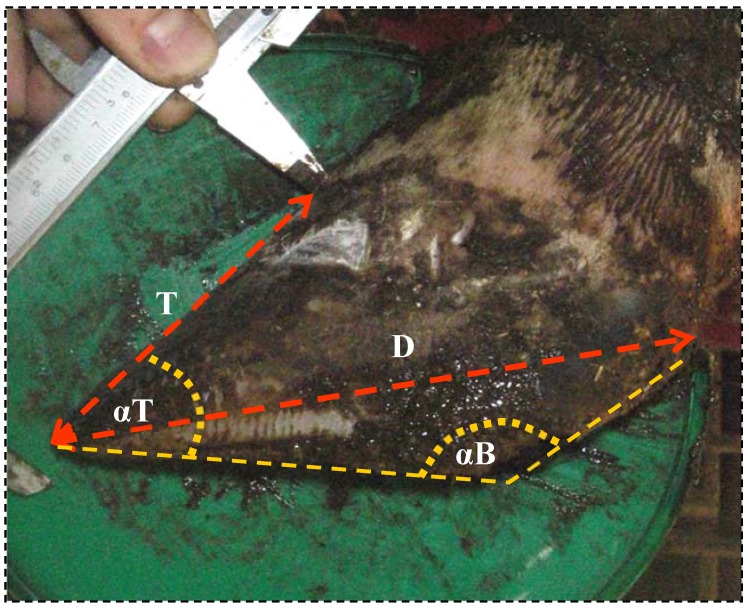
Claw measurements assessed digitally from a photo from a lateral view of left rear claw: **T**—Toe length, **D**—Diagonal, **αT**—Toe angle, **αB**—Bulb angle.

The following measurements were made (Figure 2):
Toe length:From tip of the toe to perioplium along the dorsal toe wallToe angle:The angle between the dorsal wall and sole levelDiagonalFrom tip of the toe to the most proximal part of the rear bulbBulb angle:The angle between sole level and bulbWear:Difference between present and initial distance from toe tip to brand markGrowth:Difference between present and initial distance from perioplium to brand mark.

From each picture, measurements were made three times and the mean value was used.

The contact area and pressure distribution between the claw and a standardised surface (I-Scan® system, Tekscan Inc., Boston, MA, USA) were measured for 10 selected heifers, blocked for age and breed, from each group and year, according Telezhenko *et al.* [16]. The area of the pressure sensor was 246 mm × 246 mm and it had 3.202 sense elements per cm^2^ and a 1.5 mm stainless steel plate with holder and protection for the sensor grip (comprising 0.12 mm Teflon cloth (02-QSP12, Fluorweb AB, Åkersberga) and 5 mm rubber coating for protection and stabilisation against the claw). The pressure sensor was calibrated with a hydraulic press equipped with a load cell (LPM-1-PI, Bofors, max load 200 kN, error 0.76%) in accordance with the manufacturer’s instructions. Measurements were made immediately before spring trimming in April and May each year for the heifers (Year 1) when they were immobilised in the trimming chute and of the first-calving heifers (Year 2) when they were standing in the feed stalls. Three measurements were made per animal on each occasion and each measurement included 500 frames (frequency 100 frames/second). Each measurement was translated to a contact average and from these pictures data on contact area, load, average pressure and maximal pressure were retained.

All claw disorders were recorded at trimming, when the animals were immobilised in a hydraulic trimming chute (Bulbjerg, Denmark). All the claws were trimmed and all lesions observed were recorded, even if they disappeared with trimming. The most common lesions (dermatitis, heel-horn erosion, sole and white-line haemorrhages, and sole ulcers) were scored according to a four-point scale (normal = 0, slight lesion = 1, moderate lesion = 2, severe lesion = 3).

Leg lesions were recorded for each leg at trimming and thereafter monthly according to a three-point scale for each parameter (hairlessness, swelling and ulceration) as follows: *Hairlessnes*s: Normal = 0; hairlessness less than 10 cm = 1; hairlessness more than 10 cm = 2. *Swelling*: Swelling palpable, but not visible = 0; swelling clearly visible = 1. *Ulceration*: Normal = 0; small ulcerative skin lesion (<2 cm) = 1; larger ulcerative skin lesion (>2 cm) = 2.

### 3.4. Locomotion

The locomotion of first-calving heifers was observed and scored once monthly on the same concrete alley, according to a subjective scale described by Tranter and Morris [17] where: Normal gait = 0; slight aberration of gait = 1; slight lameness = 2; moderate lameness with asymmetrical shortened steps and head bob = 3; severe lameness, difficulty moving, animal refuses to put weight on or several legs = 4.

### 3.5. Statistical Analysis

The effect of flooring system on claw conformation, claw growth and claw wear was analysed using General Linear Model (GLM, JMP 5, SAS Inst). The factors included in the model for heifers were: Flooring system (Hard 1, Soft 1); heifer cohort (2004, 2005); and interaction between cohort and flooring system, breed (SH, SR), age (months). The factors included in all models for first calvers were: Flooring system for heifers (cubicles, deep straw bedding); flooring system for first-calving heifers (slatted concrete, slatted rubber); interaction between the flooring system for recruitment heifers and first-calving heifers (Hard 1, Soft1, Hard 2, Soft 2); cohort (2004, 2005); breed (SR, SH); and days in milk (continuous). The results are presented as least squares means (LSM) and standard error (SE), with statistical significance set at *p* < 0.05.

Pressure measurements were analysed with a mixed general linear model (GLM, JMP 6, SAS Inst) that included the factors: Flooring system for recruitment heifers (Hard 1, Soft 1); flooring system for first-calving heifers (Hard 2, Soft 2); interaction between the flooring system for recruitment heifers and first-calving heifers; cohort (2004, 2005); breed (SR, SH); and days in milk (categorical variable: <150 DIM, >150 DIM).

Individual animals were randomly nested in flooring system, breed, cohort and days in milk.

In all models for contact area, mean pressure and maximal pressure corrections were made for total pressure. The results are presented as LSM and SE.

The statistical analysis of claw health included dermatitis, heel-horn erosion, sole and white-line haemorrhages, and sole ulcers. The prevalence of other claw disorders (white-line lesion, double sole, interdigital hyperplasia and warts) were too low to be analysed. Moreover, because the prevalence of sole ulcers were low, they were analysed together with severe sole haemorrhage to increase the power, as these disorders are correlated with each other [6]. Dermatitis, heel-horn erosion, sole and white-line haemorrhages were dichotomised (normal/subclinical: 0, 1, 2 or diseased/clinical 3) and analysed on the animal level. The effect of different flooring systems on claw lesions was analysed with multiple logistic regression (JMP 5, SAS Inst). The model for claw health of recruitment heifers included the factors: Flooring system (Hard 1, Soft 1); cohort (2004, 2005); interaction between cohort and flooring system; and breed (SR, SH).

In all models for claw health in first-calving heifers, corrections were made for the effect of the factors: Flooring system for heifers (Hard 1, Soft 1); flooring system for first-calving heifers (Hard 2, Soft 2); interaction between the flooring system for heifers and first-calving heifers; cohort (2004, 2005); breed (SR, SH); and days in milk (continuous variable).

For the analysis of leg lesions and locomotion only maximum values were used for each leg lesion, while lameness records for the whole housing season were used. Statistical analysis of leg lesions and locomotion was by multiple regression analysis (as for analysis of claw health). The logistic regression coefficient was transformed to Odds Ratio (OR) for first-calving heifers on concrete slats in comparison with slatted rubber mats, with a 95% confidence interval calculated for each OR.

## 4. Results

### 4.1. Impact of Flooring System on Claw Conformation in Recruitment and First-Calving Heifers

Data on claw conformation, claw wall wear and growth in various housing systems for recruitment heifers are presented in Table 1. Heifers kept in deep straw bedding (Soft 1) had significantly (*p* < 0.001) longer toes, smaller toe angle, shorter diagonal and higher bulb angle (corresponding to low heels) than heifers housed in cubicles (Hard 1). Comparison of wear and growth of the claw wall showed that both growth and wear were highest in heifers in Hard 1. Wear was slightly higher than growth, resulting in negative net growth of the toe. In Soft 1, both wear and growth were lower.

**Table 1:** Impact of flooring system on recruitment heifer claw conformation (toe length, toe angle, diagonal, bulb angle), claw wear and growth.

Conformation in Heifers	Flooring System				*p*-value *(F-test) Hard 1 and Soft 1*
	Hard 1 (*N* = 70)		Soft 1 (*N* = 68)		
	LSM	SE	LSM	SE	
Toe length, mm	70.19	0.63	79.85	0.62	<0.001
Toe angle, °	55.50	0.53	48.45	0.52	<0.001
Diagonal, mm	121.61	0.82	133.82	0.81	<0.001
Bulb angle, mm	137.38	0.67	143.38	0.66	<0.001
Wear, mm/month	6.08	0.18	2.09	0.18	<0.001
Growth, mm/month	5.46	0.22	3.54	0.22	<0.001

Claw wear was significantly higher in first-calving heifers on concrete slatted floors (Hard 2) than in those on slatted rubber mats (Soft 2). First-calving heifers that came from Hard 1 had a tendency for higher claw growth than those from Soft 1, but the differences were not significant (Table 2).

Breed significantly (*p* = 0.003) affected toe length in first-calving heifers (88.56 ± 0.63 for SH and 91.71 ± 0.81 for SR).

**Table 2:** Claw conformation and claw wall growth and wear in first-calving heifers on slatted concrete (Hard 2) or slatted rubber mats (Soft 2) coming from either cubicles (Hard 1) or deep straw bedding (Soft 1) as heifers. (**a**) Absolute values and (**b**) Significance levels.

**Claw Conformation in First-Calving Heifers (a)**	**Animal Flow**							
	**Soft 1 → Hard 2 (*N* = 29)**		**Hard 1 → Hard 2 (*N* = 27)**		**Soft 1 → Soft 2 (*N* = 24)**		**Hard 1 → Soft 2 (*N* = 31)**	
	LSM	SE	LSM	SE	LSM	SE	LSM	SE
Toe length, mm	88.86	0.97	89.83	1.07	90.09	1.02	91.77	0.95
Toe angle, °	50.65	0.79	50.82	0.87	49.04	0.83	49.66	0.77
Diagonal, mm	147.05	1.43	148.50	1.57	148.21	1.49	150.67	1.39
Bulb angle, mm	136.74	1.07	138.73	1.17	139.08	1.11	136.88	1.04
Wear, mm/month	1.46	0.11	1.27	0.12	0.98	0.12	0.84	0.11
Growth, mm/month	3.97	0.28	4.47	0.30	3.79	0.29	4.20	0.27
**Claw Conformation in First-Calving Heifers (b)**	**Effect of Flooring System and Combination of Systems. *p*-value by F-test**							
	**Year 1**		**Year 2**		**Interaction between system Year 1 and Year 2**			
Toe length, mm	0.18		0.12		0.71			
Toe angle, °	0.62		0.09		0.77			
Diagonal, mm	0.18		0.26		0.73			
Bulb angle, mm	0.92		0.82		0.06			
Wear, mm/month	0.16		<0.001		0.81			
Growth, mm/month	0.11		0.43		0.88			

### 4.2. Weight and Pressure Distribution on Claws of Recruitment Heifers in Different Flooring Systems

All parameters studied regarding pressure relations between claw and floor differed significantly between recruitment heifers kept in Soft 1 and Hard 1 (Table 3). Heifers in Hard 1 had a larger contact area, lower average contact pressure and lower maximum point load (vertical ground reaction force), expressed as an absolute value or as a percentage of total load.

**Table 3:** Contact area, average contact pressure and maximum point load between claw and a standardised surface, measured on heifers kept in free cubicles (Hard 1) or deep straw bedding (Soft 1).

Housing System	Contact Area cm^2^		Average Contact Pressure N/cm^2^		Maximum Point Load. N		Maximum Point Load. %	
	LSM	SE	LSM	SE	LSM	SE	LSM	SE
Hard 1, N = 21	32.59	1.58	35.69	1.99	99.30	12.07	10.36	0.89
Soft 1, N = 20	26.82	1.46	42.82	1.84	150.06	11.17	14.46	0.82
*P*-value *	0.01		0.01		0.003		0.001	

* Difference Hard 1, Soft 1 (F-test).

**Figure 3:**
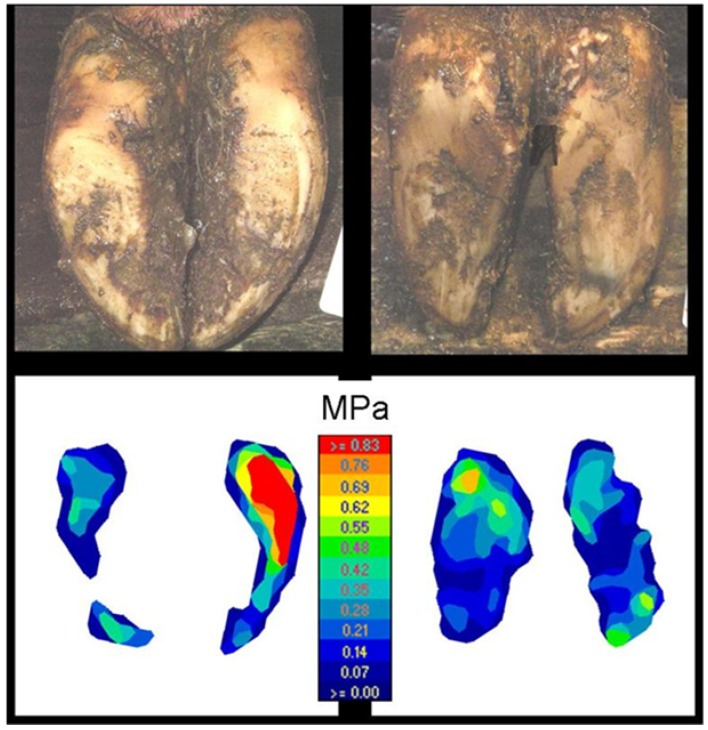
Colour-coded output from pressure plate (I-Scan^™^) from claws of recruitment heifers in deep straw bedding (Soft 1 left) and cubicles (Hard 1 right).

**Figure 4:**
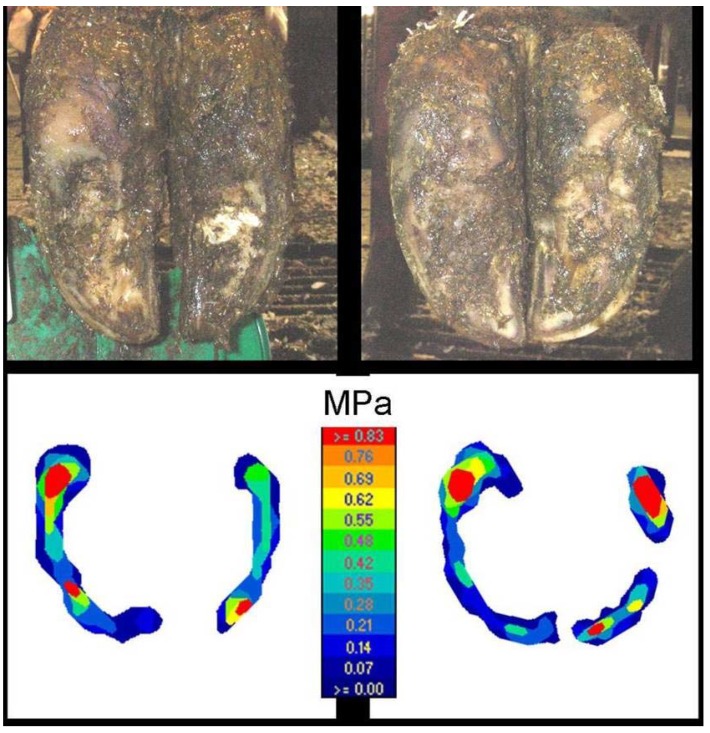
Colour-coded output from pressure plate (I-Scan™) from claws of first-calving heifers in deep straw bedding (**left**) and cubicles (**right**).

Assessment of colour-coded images from the pressure plate revealed that the wall and bulb bore the majority of the load of heifers in Soft 1, while the weight was distributed evenly over the sole contact surface of heifers in Hard 1 (Figure 3). On the other hand, no difference in loading parameters was found between first-calving heifers in Hard 2 and Soft 2, irrespective of whether the recruitment heifers were on Hard 1 or Soft 1 or of the weight distribution on the sole (Figure 4).

### 4.3. Influence of Hard or Soft Flooring System on Leg Lesions in Recruitment and First-Calving Heifers

Recruitment heifers on deep straw bedding (Soft 1) were free from all leg lesions except for one animal in cohort 2. Heifers in cubicles (Hard 1) had some mild hairlessness, but no significant difference was found between the Hard 1 and Soft 1 groups.

Most first-calving heifers had mild hairlessness. Swelling was rare and only three animals had visible swelling. Soft 2 had lower odds of ulceration (*p* = 0.02) than Hard 2 (Table 4). The proportion of animals with ulceration was small and only one first-calving heifer in Soft 2 (Hard 1 as heifer) had severe ulceration. Although the flooring system for recruitment heifers did not have a significant effect on leg lesions in first-calving heifers, animals coming from Soft 1 generally had less leg disorders than those from Hard 1.

Breed had a significant effect on the occurrence of swellings, with SH having higher odds of swelling than SR (OR = 4.99; IC 95% = 1.20–34.68; P_LR_ = 0.03).

**Table 4:** Effect of different flooring systems on leg lesions in first-calving heifers (by multiple logistic regression). Odds ratio (OR), 95% confidence interval (CI) and the results of the likelihood ratio test (P_LR_) for hairlessness, swelling and ulceration. Slatted concrete floors (Hard 2) compared with slatted rubber mats (Soft 2). *N* = 118.

Leg lesion	OR	CI 95%	P_LR_
Hairlessness	2.37	0.64–10.27	0.21
Swelling	2.45	0.73–9.21	0.15
Ulceration	2.57	1.16–5.88	0.02

### 4.4. Claw Disorders of Recruitment and First-Calving Heifers

**Figure 5:**
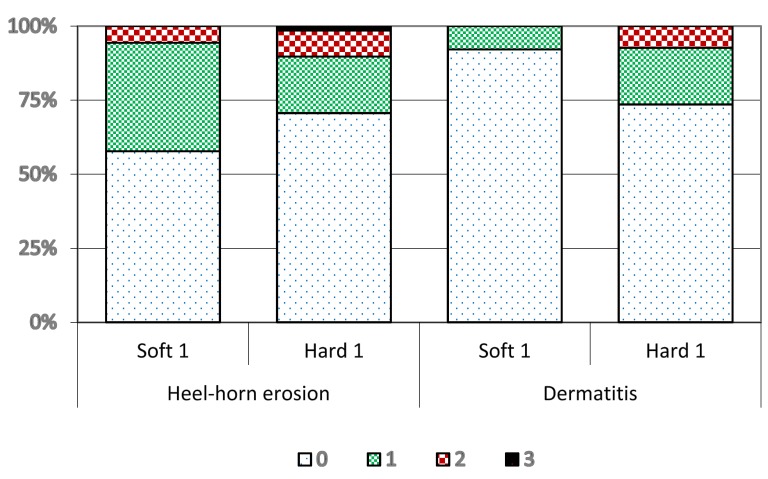
Prevalence of heel-horn erosion and dermatitis of different severity (0 = none, 1 = mild, 2 = moderate, 3 = severe) in recruitment heifers at spring trimming after Year 1 (cohort 1 and 2) in different housing systems (cubicles (Hard 1) or deep straw bedding (Soft 1)).

**Figure 6:**
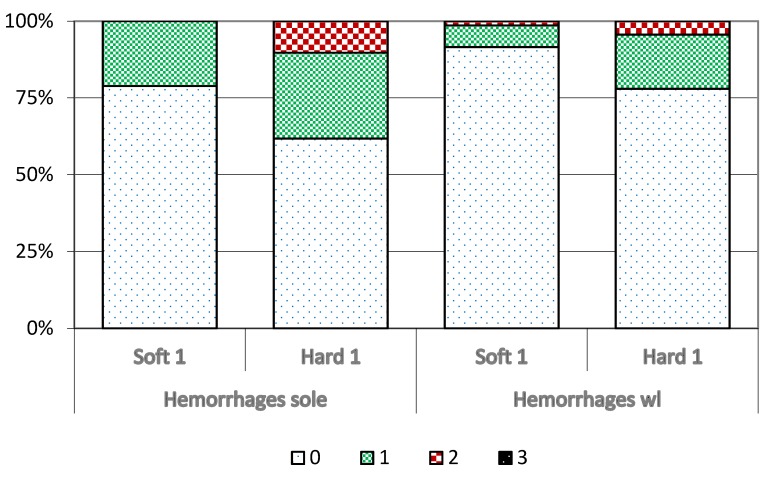
Prevalence of sole and white-line haemorrhages of different severity (0 = none, 1 = mild, 2 = moderate, 3 = severe) in recruitment heifers at spring trimming after Year 1 (cohort 1 and 2) in different housing systems (cubicles (Hard 1) or deep straw bedding (Soft 1).

Data on hygiene- (dermatitis and heel-horn erosion) and laminitis-related (sole haemorrhages including sole ulcer and white-line haemorrhages) claw disorders of recruitment heifers at spring trimming after being housed in Year 1 (cohorts 1 and 2) are presented in Figure 5 and Figure 6 and Table 5. Heifers in Soft 1 had a higher total prevalence (*p* = 0.06) and a higher proportion of mild heel-horn erosion, but Hard 1 heifers had a higher proportion of moderate and severe heel-horn erosion. Heifers in Soft 1 were virtually free from dermatitis, while 26% of heifers in Hard 1 had mild and moderate dermatitis (*p* < 0.002).

Moreover, 28% of heifers in Hard 1 had mild and 10% had moderate sole haemorrhages, compared with 21% of heifers with mild sole haemorrhages in Soft 1 (*p* = 0.06). There was no significant difference in white-line haemorrhages between heifers in Soft 1 and Hard 1.

**Table 5:** Effect of different flooring systems on claw disorders of recruitment heifers in Year 1 after the housing season before grazing (by multiple logistic regression). Odds ratio (OR), 95% confidence interval (CI) and the results of the likelihood ratio test (P_LR_) for heel-horn erosion, dermatitis and haemorrhage of the sole and white line. Deep straw bedding (Soft 1) compared with cubicles (Hard 1). *N* = 139.

Claw Disorder (Prevalence of any Severity)	OR	CI 95%	P_LR_
Heel-horn erosion	2.15	0.97–4.93	0.06
Dermatitis	0.33	0.12–0.82	0.02
Sole haemorrhages *	0.46	0.21–1.02	0.06
White-line haemorrhages	0.44	0.11–1.58	0.20

* Haemorrhages of the sole + sole ulcers.

### 4.5. Effect of Grazing on Claw Disorders at Autumn Trimming in First-Calving Heifers

Analysis of claw disorders at autumn trimming after grazing and after being housed in first-calving heifers (Year 2) showed no significant differences between heifers from either Hard 1 or Soft 1. The prevalence of heel-horn erosion and dermatitis was lower in heifers from Soft 1 than from Hard 1. However, not all heifers calved at the estimated time, which reduced the number of animals in the analysis. Heel-horn erosion declined sharply in animals, which had previously been on deep straw bedding, while the prevalence of dermatitis was about the same after grazing as before. However, slightly more first-calving heifers had severe dermatitis than recruitment heifers. The prevalence of sole and white-line haemorrhages was higher after grazing compared with before grazing and the proportion of severe sole haemorrhages and sole ulcers was higher in animals, which came from Hard 2 than in those, which came from Soft 1 before grazing.

### 4.6. Effect of Different Flooring Systems on Claw Disorders at Spring Trimming in First-Calving Heifers

The prevalence and severity of all claw disorders in first-calving heifers (cohort 1 and cohort 2) increased after the housing season (Year 2) compared with the previous results from the recruitment heifers (Year 1) and after the grazing between Year 1 and Year 2, regardless of flooring system. First-calving heifers on slatted concrete (Hard 2) had a lower prevalence of heel-horn erosion than those on slatted rubber (Soft 2), regardless of their flooring system as recruitment heifers during Year 1. First-calving heifers on Soft 2, which came from Hard 1, had more heel-horn erosion (44% mild and moderate 6%) than those that came from Soft 1 (34% mild and 7% moderate).

First-calving heifers on Hard 2 that came from Soft 1 had a higher prevalence of dermatitis (17% mild and 13% moderate) than those which came from Hard 1 (11% mild and 11% moderate). This relationship was reversed in heifers on Soft 2, where heifers from Soft 1 had less dermatitis (17% mild and 3% moderate) than those from Hard 1 (16% mild 13% moderate and 3% severe).

**Figure 7:**
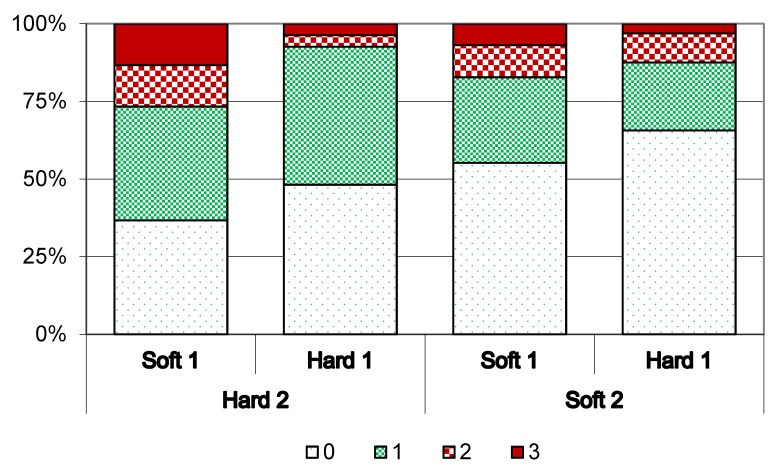
Prevalence of sole haemorrhages (including sole ulcers) of different severity score (0 = none, 1 = mild, 2 = moderate, 3 = severe) in first-calving heifers (cohort 1 and 2) at spring trimming in Year 2 in different flooring systems (slatted concrete (Hard 2) or slatted rubber mats (Soft 2)) coming from either deep straw bedding (Soft 1) or cubicles (Hard 1).

Haemorrhages of the sole were most common and most severe in the first-calving heifers in Hard 2, which came from Soft 1 (Figure 7) and only 37% of these animals were free from sole haemorrhages. Among first-calving heifers on Hard 2 coming from Hard 1, sole haemorrhages were less prevalent and less severe (44% mild and 4% moderate and severe). First-calving heifers on Soft 2 and coming from Hard 1 had the lowest prevalence of sole haemorrhages (22% mild, 9% moderate and 2% severe).

**Figure 8:**
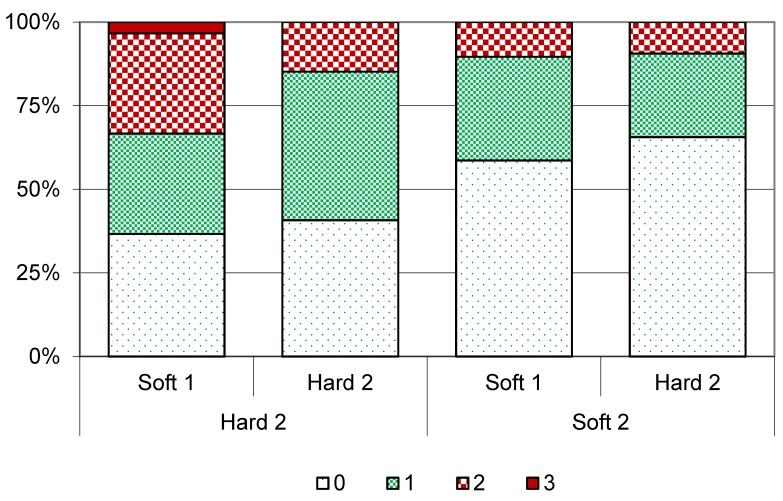
Prevalence of white-line haemorrhages of different severity score (0 = none, 1 = mild, 2 = moderate, 3 = severe) in first-calving heifers (cohort 1 and 2) at spring trimming in Year 2 in different flooring systems (slatted concrete (Hard 2) or slatted rubber mats (Soft 2)) coming from either deep straw bedding (Soft 1) or cubicles (Hard 1).

**Table 6:** Effect of different flooring systems on claw disorders in first-calving heifers in Year 2 after the housing season (result of multiple logistic regression). Odds ratio (OR), 95% confidence interval (CI) and the results of the likelihood ratio test (P_LR_) for heel-horn erosion, dermatitis and sole and white-line haemorrhages. Slatted concrete floors (Hard 2) compared with slatted rubber mats (Soft 2). *N* = 118.

Claw disorder	OR	CI 95%	P_LR_
Heel-horn erosion	0.49	0.22–1.09	0.08
Dermatitis	1.06	0.44–2.52	0.89
Sole haemorrhages *	2.19	1.00–4.97	0.05
White-line haemorrhages	2.82	1.28–6.43	0.01

* Also including sole ulcers.

The prevalence of white-line haemorrhages showed the same pattern as for sole haemorrhages, *i.e.*, the highest prevalence was seen in first-calving heifers that came from Soft 1 to Hard 2 and the lowest prevalence and severity in animals that came from Hard 1 to Soft 2 (Figure 8).

There were some significant differences in effect of flooring system at spring trimming in Year 2 on first-calving heifers (Table 6). The OR of sole haemorrhages was over twice as high and the OR of white-line haemorrhages almost three times higher for first-calving heifers in Hard 2 compared with Soft 2. Animals in Hard 2 had a tendency for lower OR of heel-horn erosion in comparison with Soft 2. No significant effect of the flooring system on dermatitis was found.

Effect of breed was significant for the prevalence of sole haemorrhages, with SH having higher OR of sole haemorrhages than SR at spring trimming in Year 2 (OR = 2.62; IC 95% = 1.11–6.48; P_LR_ = 0.03).

### 4.7. Locomotion Scoring

Only a few animals showed clinical lameness, and animals in Hard 2 had significantly higher locomotion scores (OR = 3.64; IC 95% = 1.33–11.09; P_LR_ = 0.01) than those in Soft 2 (Table 7), irrespective of whether they came from Soft 1 or Hard 1. First-calving heifers in Hard 2 coming from Hard 1 had higher locomotion scores than those from Soft 1.

**Table 7:** Locomotion scoring (% distribution within group) in first-calving heifers on slatted concrete (Hard 2) or slatted rubber mats (Soft 2), but reared in cubicles (Hard 1) or deep straw bedding (Soft 1) as recruitment heifers.

***Year 2***	**Hard 2. *N* = 57**						**Soft 2. *N* = 61**					
Year 1	Hard 1. *N* = 27			Soft 1. *N* = 30			Hard 1. *N* = 32			Soft 1. *N* = 29		
Severity	0	1	2	0	1	2	0	1	2	0	1	2
Locomotion	66.6	26.0	7.4	76.7	20.0	3.3	87.5	12.5	0	89.7	6.9	3.4

## 5. Discussion

Management and housing in the transition period is known to be highly important for future production and health in dairy cows. The importance of conditions provided for recruitment heifers on their future performance does not receive much attention in the literature. However, already from the beginning of gestation pregnant heifers have to adapt to both physiological and management changes. It is thus important to supervise their management not only during the transition period but also throughout their gestation. The experimental 2 × 2 factor layout used in this study aimed to compare the effects of different flooring systems for heifers in the year before and the year after calving during two consecutive housing seasons.

Unfortunately, not all the initial hypotheses in this study could be tested given the statistical power of the study. The somewhat lower frequency of lesions in this study could be explained by the extended grazing period, frequent claw trimming and the organic production regime. A higher frequency of lesions could be expected under more intensive production conditions, less claw care and with zero grazing. However, it is still important to describe the observed biological effects of housing of recruitment heifers for future feet and leg health in first-calving heifers.

### 5.1. Claw Conformation and Pressure Distribution

In contrast to hereditary conformation, the effects of wear and growth on claw horn determining conformation are highly dependent on the abrasiveness of the walking areas, animal activity and claw trimming [18,19,20]. In the present study, heifers in cubicles (Hard 1) had less overgrown claws and more sole haemorrhages than those on deep straw bedding (Soft 1). This is in agreement with Vermunt and Greenough [14], who found that heifers on concrete slats indoors had less overgrown claws and more sole lesions than those on a dry lot outdoors. While the conformation regarding all measurements was theoretically significantly better in Hard 1 than in Soft 1, sole lesions were more prevalent in cubicles with concrete alleys. This exemplifies two different relationships between conformation and claw disease depending on the cause of the conformation and the aetiology of claw disease. One is the lack of wear and of regular trimming, which increases the risk of overgrowth and uneven weight distribution and of undetected claw horn lesions, respectively. The other is too much wear, thinner soles and increasing risk of exposing the corium to trauma and claw disease. In our study, the lying surface in Hard 1 and Soft 1, although not studied, possibly influenced the lying time and exposure of claws to the concrete alleys, contributing to the different results between Hard 1 and Soft 1. As a result of greater wear of the claw wall in Hard 1, the feet of those heifers had a larger contact surface and a higher load on the sole than heifers in Soft 1. It is likely that fine vessels in the sole corium were affected and heifers in Hard 1 had a higher prevalence and more severe sole haemorrhages than heifers in Soft 1. Heifers in Soft 1 had a higher wall and thus a higher maximum point load on the bearing surface and less on the sole area.

Claw horn growth of first-calving heifers did not differ between the groups (Hard 2 and Soft 2). However, there was a tendency for animals reared in Hard 1 to conserve their higher horn growth rate over the pasture period and through their first lactation, which is remarkable. Although there were no significant differences in claw conformation between first-calving heifers, there was slightly (about 1 mm) higher claw horn wear in Hard 2 than in Soft 2. This is in agreement with Telezhenko *et al.* [18] and Vanegas *et al.* [21], who found reduced wear on rubber flooring compared with concrete in identical cubicles. Vokey *et al.* [19] found lower net growth in cows housed on rubber floors compared with concrete over a 15-week study period. However, they compared many different combinations of alley flooring and cubicle bedding and the differences were small.

The small differences in claw conformation between Hard 2 and Soft 2 indicate that there were no significant differences in pressure distribution in the first-calving heifers. Unlike heifers kept in Hard 1 with alleys of new concrete, Hard 2 animals that walked on the worn slatted concrete had a relatively small contact area and a high pressure. The pressure was applied on the wall and bulb and the weight distribution in Hard 2 was similar to that in Soft 2.

### 5.2. Leg Lesions

The finding that heifers in deep straw bedding (Soft 1) were virtually free from leg lesions when they were examined at the end of the housing period Year 1 is not surprising. It is also not surprising that the heifers in concrete cubicles were more affected and had more severe leg lesions, especially of front leg knees (carpus). These leg injuries are to a very large extent related to the lying surface and space available, and have been highlighted in previous studies. Livesey *et al.* [22] clearly showed that cows on a straw yard had least hock injuries, followed by cows in cubicles equipped with mattresses or rubber mats. Haskell *et al.* [23] also showed fewer leg injuries in straw yards and fewer leg lesions in cows on pasture than in animals that never went out to pasture. Concrete surfaces were not included in these comparisons, but, in other studies, the preference for various surfaces other than concrete, such as mattresses and rubber mats, was always higher [24,25].

In the milking group (Year 2), all the cubicles (Hard 2 and Soft 2) were equipped with identical mattresses (KEW plus™, Kraiburg). Although these mattresses were considerably softer than the rubber mats in the heifer compartment, most first-calving heifers had a certain hairlessness and swelling. Obviously the sensitivity to leg injuries was greater in first-calving heifers, which can be associated with increased weight and increased metabolic load. It is well known that sand stalls usually produce fewer and less serious hock lesions than rubber chip mattresses [19,26]. Despite the large difference in the leg health of heifers and although a lower frequency was seen in animals that came from deep straw bedding in our study, it did not result in any statistically significant difference regarding the previous rearing system of the heifers. It is likely that the long grazing period between the two housing periods studied levelled out any effects. Regardless of previous flooring system, first-calving heifers on slatted concrete floors (Hard 2) had more hairlessness and swelling, and significantly more ulcers, than those on slatted rubber mats (Soft 2). This finding highlights the importance of exposure time for leg injuries, because all the animals had exactly the same bedding. Thus, the effect was indirectly caused by the alley flooring. Either the animals were lying more because of sore claws owing to the higher prevalence of haemorrhages and ulcers of the claws, or lying less and with shorter lying bouts because of higher comfort with rubber in the alleys reducing the risk of leg injuries. It has previously been shown that the activity and social behaviour of cows in rubber alleys is increased compared with that of cows in concrete alleys [27,28].

### 5.3. Claw Disorders

In the present study, the trimming itself was a method of assessing claw disorders and at the same time certainly confounded the results, because trimming is a measure to improve conformation and to prevent claw disorders [20]. Thus, the heifers’ feet were trimmed regularly three times during the study, plus at the final examination after their first lactation, which almost certainly reduced the risk of claw disorders appearing and developing. However, there were significant differences in claw health between different flooring systems, even if only weak significant differences could be detected in some comparisons.

There was a higher prevalence of dermatitis in Hard 1 than in Soft 1. The ability to maintain good hygiene is a key attribute in a dairy house, with hygiene of alleys in particular directly correlated to foot hygiene and infectious claw disorders. The accumulation of manure and urine increases ammonium emissions and creates good conditions for the growth of bacteria that cause claw diseases [29,30]. In addition to the increased risk of infections, because the cows have to walk and stand in manure, the claw horn and skin is softened and becomes more vulnerable. In scraped alleys, the risk of encountering urine mixed with manure, which increases release of ammonia, is higher. If urine can be separated from manure by sloping or slatted flooring, the floor environment will improve, as will foot hygiene and hygiene-related claw disorders [31]. In Hard 1, heifers had to walk and stand on the non-drained, concrete alleys for a longer period than in Soft 1 in order to find their cubicle. With more exposure to urine and manure on walking surfaces, the risk of dermatitis increases and therefore also the risk of heel-horn erosion, which usually arises secondary to dermatitis [29,32]. Dermatitis disturbs the formation of new heel horn from the coronary band and is to some extent keratinolytic on the surface of the claw horn [33].

Because dermatitis was less prevalent in Soft 1, it was surprising that heel-horn erosion was more prevalent in Soft 1 than in Hard 1 after the housing season. A previous study [34] also reported more heel-horn erosion in heifers in a straw yard compared with cubicles, but dermatitis was not recorded. When the dermatitis heals, spontaneously or by some intervention, new skin is rapidly formed. However, when the heel horn starts to recover, it takes months for old cracks to be replaced with new heel horn. Therefore, heel-horn erosion can persist for a long time after dermatitis has disappeared. Moreover, the faster the horn grows, the faster the erosions recover, provided that no new dermatitis develops. The heel-horn erosion could have persisted longer on deep straw bedding because of less wear and lower net growth compared with Hard 1, where the heel-horn erosion might have been worn away and been replaced more quickly because of the higher wear and growth rate. After the grazing period, the prevalence of dermatitis and heel-horn erosion decreased in all animals and did not differ between groups, which is consistent with earlier studies [35].

First-calving heifers walked on slatted floors except for the twice daily stay in the milking area. The drainage of urine and manure from slatted floors is dependent on drainage area, manure consistency, cow traffic, amount of litter on the floors *etc.* In the present study, heel-horn erosion was more prevalent in first-calving heifers in Soft 2, although dermatitis was equally prevalent as in Hard 2. Vanegas *et al.* [21] also recorded more heel-horn erosion in cows with rubber mat alleys than on concrete. However, if heel-horn erosion was established in the early housing period in animals of both groups, the lesions can possibly have been conserved longer, until spring trimming in animals on rubber with less wear than on concrete. That is the same explanation as for the recruitment heifers. Another explanation is reasonable in this study. When covering the original concrete slats with slatted rubber mats, the drainage area was reduced from 20% to 15% because of the reinforced construction and by reducing openings from 4 cm to 3.5 cm. Thus, the drainage of manure decreased, hygiene was impaired and the risk of heel-horn erosion increased. Using scraping robots or scraping devices available on the market could easily solve this problem.

The higher prevalence of sole haemorrhages in Hard 1 and the connections with conformation and weight bearing have already been discussed. Haemorrhages of the sole horn are a sign of erythrocytes and serum leaking from the capillary bed in the corium, which is incorporated in the growing horn and reaches the sole surface 2–3 months later. If the protective wall is worn down by longer exposure to abrasive flooring or over-trimming, a higher load is directed to the sensitive horn-producing sole corium, causing traumatic injury [16]. White-line haemorrhages have a somewhat different development and are considered to appear some weeks before sole haemorrhages [36,37]. The laminitis-related lesions and related damage are also strongly related to hormonal, metabolic and management changes around calving [38,39]. As diets, feeding and any management practice other than the flooring system were identical between the experimental groups, it is most likely that these detected differences between soft and hard floorings had a biomechanical/traumatic background. The higher wall and thus a higher maximum point load on the bearing surface in Soft 1 heifers might have resulted in more damage of the white line. However, the hoof wall is the strongest part of the claw and can withstand a heavy load in a vertical direction. Lesions and cracks of the white line are the result of poor quality horn produced from the lamellar corium and can result in white-line ulceration and abscess due to the oblique strain that arises when animals turn on the loaded claw. For animals on deep straw bedding this risk was minimised because all rising and lying was on soft ground and the claws were only exposed to concrete during feeding. The larger point load that was recorded for heifers in Soft 1 was highly probably applied on the bearing surface of the wall and was therefore dispersed on a large area of lamellae (representing the wall and bearing surface), which reduced the risk of sole haemorrhages.

The results from spring claw trimming give a retrospective view of the claw disorders during the housing season for the two experimental groups Hard 2 and Soft 2 and, indirectly, the previous experimental groups Hard 1 and Soft 1. The two flooring systems seemed to have a far greater impact on the claw health of the first-calving heifers than of the recruitment heifers, as also shown by Chaplin *et al.* [40] and discussed above. Bergsten and Frank [41] tried to provoke sole haemorrhages with only high concentrate feeding of pregnant heifers, without any significant results. On the other hand, the same animals reacted significantly more on similar diets after calving [42]. The present study clearly showed that the slatted rubber flooring resulted in lower prevalence of sole and white-line haemorrhages. While Bergsten and Frank [42] saw a smaller increase of sole haemorrhages in tied first-calving heifers on rubber compared with concrete, it is difficult to judge whether this was a result of less exposure due to more comfortable lying or less traumatic influence of rubber when standing compared with concrete. Other studies have presented results confirming that comfortable bedding reduces the risk of claw disorders and lameness [10,43]. Thus, the present study more clearly confirms that rubber itself reduced the risk of sole and white-line haemorrhages (including sole ulcers) because cows are more likely to stand for longer on rubber than on concrete [27,44,45] and in this study the bedding was exactly the same. A previous study [46] showed that the prevalence of sole and white-line haemorrhages was lower in first-calving heifers that were kept on a straw yard also after calving than in cubicles and concrete alleys (irrespective of bedding type). If the introduction to a hard surface can be avoided or delayed in connection with calving, the risk of sole and white-line haemorrhages, sole ulcers and lameness can be reduced. Spring calving heifers that were adapted to concrete floors during a longer time period thus had a significantly lower increase in sole haemorrhages than those that came directly from pasture to concrete floors [42]. Boyle *et al.* [47] found no difference in claw disorders between cows on rubber or concrete alleys, but cows on concrete alleys stood more in the soft stalls, which was suggested to have the same relieving effect as standing on rubber in the alleys. In other contexts, it has been shown that to avoid sole ulcers and sole haemorrhages, the floor should be slightly shock absorbing and slip resistant [48,49]. As the transition period is of special concern for changes of flooring during this time, such changes should be avoided if possible. Webster [50] clearly showed that keeping heifers in a straw yard until eight weeks after calving, compared with moving them to cubicles with concrete flooring four weeks before calving, significantly reduced the severity of sole haemorrhages and sole ulcers. However, it is unclear how the young stock was managed in that study. Because the exposure time for the different floors was not assessed in the present study and because all animals had rubber in the feed stalls in front of the feeding platform, it is likely that the differences between Soft 2 and Hard 2 were even larger than revealed. Furthermore, our earlier studies have shown that if cows are given the opportunity to choose, the majority choose softer flooring (rubber mat) over harder (concrete) flooring, both when walking and standing [51].

### 5.4. Influence of Recruitment Heifer Floorings on Claw Disorders and Locomotion in the Milking Herd

The intention of this study was to determine whether previous rearing of heifers on soft or hard flooring systems affected claw health and locomotion at their first lactation. It has long been a clinical observation that adapting heifers to their first lactation is of great importance, although adapting to new flooring several weeks ahead is not enough [50,52]. In both Soft 2 and Hard 2, the prevalence and severity of haemorrhages of the sole and white line were higher when the heifers came from Soft 1 compared with Hard 1, confirming our hypothesis. On the other hand, the heifers with the least number of haemorrhages and the least severity were those coming from Hard 1 and calving in Soft 2. It seems as though a traumatic claw challenge by hard flooring, resulting in sole haemorrhages during the rearing period, was beneficial for heifers after calving. This could be because heifers were able to cope with disorders while not subjected to the challenges of the transition period. If the animals obtain their first experience of hard floors during the transition period, their chances to cope with laminitis-related disorders are much lower, which explains the higher prevalence of these lesions in heifers reared on straw beds (Soft 1) and moved to concrete alleys (Hard 2) than in those moved from Hard 1 to Soft 2 flooring. For different reasons, the hypothesis could not be fully explored. First, all heifers were grazed between the housing seasons and the time for grazing was extended on the organic experimental farm, which would have equalised adaptation. Secondly, the claws were trimmed rather frequently and abnormal conformation with overgrowth was corrected, which would have equalised weight distribution for both Soft 1 and Hard 1. Thirdly, the study farm normally had at least a three-week adaptation period to the dairy house, which was the same for both groups. Therefore, preventive trimming to give a claw conformation with optimal weight distribution and a long-term adaptation avoiding changing from soft to hard flooring during the transition period could be advised to reduce the risk of trauma-related sole lesions for first-calving heifers.

Although no severe lameness was detected, locomotion was significantly less affected in first-calving heifers on slatted rubber floors than on slatted concrete floors. Vanegas *et al.* [21] also found less lameness on solid rubber flooring compared with solid concrete when cubicles were identical. Because lameness is usually a result of claw injuries [53,54] and more disorders of the sole and white line were seen on the concrete floors, these lesions may certainly explain this difference. Heel-horn erosion normally does not lead to lameness and there was no difference in the prevalence of dermatitis between the two groups of first-calving heifers. The leg lesions (hock ulcers) could also have contributed to higher locomotion scores. However, cause-effect is not clear and the leg lesions could equally well be explained by a longer lying time due to poor locomotion. Haskell *et al.* [23] found a higher prevalence of lameness and leg lesions in animals that were zero-grazed than in those which were grazed, and in cows housed in free stalls with concrete alleys than in those housed in straw yards. All the cows in the present study were grazed for four months and all cows had feed stalls with rubber mats, both of which are preventive measures for lameness.

## 6. Conclusions

The tougher cubicle system with a larger area of concrete flooring for recruitment heifers resulted in more wear, shorter claw length and a higher prevalence of sole disorders compared with those housed on deep straw bedding. After calving, the heifers housed on slatted rubber mats had less lameness and leg lesions (hock ulcers), probably because of lower prevalence of sole and white-line disorders. A higher prevalence of sole and white-line disorders was found in heifers reared on a soft and then going to a hard flooring system after calving than in those reared on a hard and then going to a soft flooring system after calving. This effect could probably have been more provocative with more abrupt changes *i.e.*, without the grazing and regular trimming that was part of the study design.

## References

[1] Tranter W.P., Morris R.S. (1992). Hoof Growth and Wear in Pasture-Fed Dairy-Cattle. N. Z. Vet. J..

[2] Politiek R.D., Distl O., Fjeldaas T., Heeres J., McDaniel B.T., Nielsen E., Peterse D.J., Reurink A., Strandberg P. (1986). Importance of claw quality in cattle: Review and recommendations to achieve genetic improvement. Report of the E.A.A.P. working group on “claw quality in cattle”. Livest. Prod. Sci..

[3] Distl O. (1999). Breeding for soundness of feet and legs in dairy cattle. Zuchtungskunde.

[4] Uggla E., Jakobsen J.H., Bergsten C., Eriksson J.-A., Strandberg E. (2008). Genetic correlations between claw health and feet and leg conformation traits in Swedish dairy cows. Interbull Bull..

[5] Gitau T., Mbiuki S.M., McDermott J.J. (1997). Assessment of bovine hoof conformation and its association with lameness, animal factors and management practices on small-scale dairy farms in Kiambu district, Kenya. Onderstepoort J. Vet. Res..

[6] Manske T., Hultgren J., Bergsten C. (2002). Prevalence and interrelationships of hoof lesions and lameness in Swedish dairy cows. Prev. Vet. Med..

[7] Ingvartsen K.L. (2006). Feeding- and management-related diseases in the transition cow—Physiological adaptations around calving and strategies to reduce feeding-related diseases. Anim. Feed Sci. Technol..

[8] Mulligan F.J., Doherty M.L. (2008). Production diseases of the transition cow. Vet. J..

[9] Leonard F.C., O’Connell J., O’Farrell K. (1994). Effect of different housing conditions on behaviour and foot lesions in Friesian heifers. Vet. Rec..

[10] Cook N.B., Bennett T.B., Nordlund K.V. (2004). Effect of free stall surface on daily activity patterns in dairy cows with relevance to lameness prevalence. J. Dairy Sci..

[11] Calderon D.F., Cook N.B. (2011). The effect of lameness on the resting behavior and metabolic status of dairy cattle during the transition period in a freestall-housed dairy herd. J. Dairy Sci..

[12] Vermunt J.J., Greenough P.R. (1996). Claw conformation of dairy heifers in two management systems. Br. Vet. J..

[13] Vermunt J.J., Greenough P.R. (1996). Hock angles of dairy heifers in two management systems. Br. Vet. J..

[14] Vermunt J.J., Greenough P.R. (1996). Sole haemorrhages in dairy heifers managed under different underfoot and environmental conditions. Br. Vet. J..

[15] Graunke K.L., Telezhenko E., Hessle A., Bergsten C., Loberg J.M. (2011). Does rubber flooring improve welfare and production in growing bulls in fully slatted floor pens?. Anim. Welf..

[16] Telezhenko E., Bergsten C., Magnusson M., Ventorp M., Nilsson C. (2008). Effect of different flooring systems on weight and pressure distribution on claws of dairy cows. J. Dairy Sci..

[17] Tranter W.P., Morris R.S. (1991). A case study of lameness in three dairy herds. N. Z. Vet. J..

[18] Telezhenko E., Bergsten C., Magnusson M., Nilsson C. (2009). Effect of different flooring systems on claw conformation of dairy cows. J. Dairy Sci..

[19] Vokey F.J., Guard C.L., Erb H.N., Galton D.M. (2001). Effects of alley and stall surfaces on indices of claw and leg health in dairy cattle housed in a free-stall barn. J. Dairy Sci..

[20] Manske T., Hultgren J., Bergsten C. (2002). The effect of claw trimming on the hoof health of Swedish dairy cattle. Prev. Vet. Med..

[21] Vanegas J., Overton M., Berry S.L., Sischo W.M. (2006). Effect of rubber flooring on claw health in lactating dairy cows housed in free-stall barns. J. Dairy Sci..

[22] Livesey C.T., Marsh C., Metcalf J.A., Laven R.A. (2002). Hock injuries in cattle kept in straw yards or cubicles with rubber mats or mattresses. Vet. Rec..

[23] Haskell M.J., Rennie L.J., Bowell V.A., Bell M.J., Lawrence A.B. (2006). Housing system, milk production, and zero-grazing effects on lameness and leg injury in dairy cows. J. Dairy Sci..

[24] Herlin A.H. (1997). Comparison of lying area surfaces for dairy cows by preference, hygiene and lying down behaviour. Swed. J. Agric. Res..

[25] Rodenburg J., House H.K. The impact of free stall base and bedding on cow comfort. Proceedings of the Dairy Housing and Equipment Systems: Managing and Planning for Profitability Conference.

[26] Weary D.M., Taszkun I. (2000). Hock lesions and free-stall design. J. Dairy Sci..

[27] Benz B. (2002). Elastic Flooring Materials for Concrete Slatted Floors in Free Stall Houses. Ph.D. Thesis.

[28] Platz S., Ahrens F., Bendel J., Meyer H.H., Erhard M.H. (2008). What happens with cow behavior when replacing concrete slatted floor by rubber coating: A case study. J. Dairy Sci..

[29] Bergsten C., Pettersson B. (1992). The cleanliness of cows tied in stalls and the health of their hooves as influenced by the use of electric trainers. Prev. Vet. Med..

[30] Rodriguez Lainz A., Hird D.W., Carpenter T.E., Read D.H. (1996). Case-control study of papillomatous digital dermatitis in Southern California dairy farms. Prev. Vet. Med..

[31] Hultgren J., Bergsten C. (2001). Effects of a rubber-slatted flooring system on cleanliness and foot health in tied dairy cows. Prev. Vet. Med..

[32] Toussaint Raven E., Haalstra R.T., Peterse D.J. (1985). Cattle Footcare and Claw Trimming.

[33] Egerton J.R., Yong W.K., Riffkin G.G. (1989). Footrot and Foot Abscess of Ruminants.

[34] Livesey C.T., Harrington T., Johnston A.M., May S.A., Metcalf J.A. (1998). The effect of diet and housing on the development of sole haemorrhages, white line haemorrhages and heel erosions in Holstein heifers. Anim. Sci..

[35] Andersson L., Lundström K. (1981). The influence of breed, age, body weight and season on digital diseases and hoof size in dairy cows. Zentralbl. Veterinärmed. A.

[36] Kempson S.A., Logue D.N. (1993). Ultrastructural observations of hoof horn from dairy cows: Changes in the white line during the first lactation. Vet. Rec..

[37] Leach K.A., Logue D.N., Kempson S.A., Offer J.E., Ternent H.E., Randall J.M. (1997). Claw lesions in dairy cattle: Development of sole and white line haemorrhages during the first lactation. Vet. J..

[38] Bergsten C. (2003). Causes, risk factors, and prevention of laminitis and related claw lesions. Acta Vet. Scand. Suppl..

[39] Tarlton J.F., Holah D.E., Evans K.M., Jones S., Pearson G.R., Webster A.J. (2002). Biomechanical and histopathological changes in the support structures of bovine hooves around the time of first calving. Vet. J..

[40] Chaplin S.J., Ternent H.E., Offer J.E., Logue D.N., Knight C.H. (2000). A comparison of hoof lesions and behaviour in pregnant and early lactation heifers at housing. Vet. J..

[41] Bergsten C., Frank B. (1996). Sole haemorrhages in tied heifers in early gestation as an indicator of laminitis: Effects of diet and flooring. Acta Vet. Scand..

[42] Bergsten C., Frank B. (1996). Sole haemorrhages in tied primiparous cows as an indicator of periparturient laminitis: Effects of diet, flooring and season. Acta Vet. Scand..

[43] Cook N.B., Nordlund K.V. (2009). The influence of the environment on dairy cow behavior, claw health and herd lameness dynamics. Vet. J..

[44] Cramer G., Lissemore K.D., Guard C.L., Leslie K.E., Kelton D.F. (2008). Herd- and cow-level prevalence of foot lesions in Ontario dairy cattle. J. Dairy Sci..

[45] Haufe H.C., Gygax L., Steiner B., Friedli K., Stauffacher M., Wechsler B. (2009). Influence of floor type in the walking area of cubicle housing systems on the behaviour of dairy cows. Appl. Anim. Behav. Sci..

[46] Laven R.A., Livesey C.T. (2004). The effect of housing and methionine intake on hoof horn hemorrhages in primiparous lactating Holstein cows. J. Dairy Sci..

[47] Boyle L.A., Mee J.F., Kiernan P.J. (2007). The effect of rubber versus concrete passageways in cubicle housing on claw health and reproduction of pluriparous dairy cows. Appl. Anim. Behav. Sci..

[48] Benz B., Wandel H., Jungbluth T. Yielding walking areas in loose house systems. Proceedings of the 12th International Symposium on Lameness in Ruminants.

[49] Ouweltjes W., van der Werf J.T., Frankena K., van Leeuwen J.L. (2011). Effects of flooring and restricted freestall access on behavior and claw health of dairy heifers. J. Dairy Sci..

[50] Webster A.J. (2002). Effects of housing practices on the development of foot lesions in dairy heifers in early lactation. Vet. Rec..

[51] Telezhenko E., Lidfors L., Bergsten C. (2007). Dairy cow preferences for soft or hard flooring when standing or walking. J. Dairy Sci..

[52] Manske T. (2002). Hoof Lesions and Lameness in Swedish Dairy Cattle: Prevalence, Risk Factors, Effects of Claw Trimming and Consequences for Productivity.

[53] Murray R.D., Downham D.Y., Clarkson M.J., Faull W.B., Hughes J.W., Manson F.J., Merritt J.B., Russell W.B., Sutherst J.E., Ward W.R. (1996). Epidemiology of lameness in dairy cattle: Description and analysis of foot lesions. Vet. Rec..

[54] Flower F.C., Weary D.M. (2006). Effect of hoof pathologies on subjective assessments of dairy cow gait. J. Dairy Sci..

